# Nutritional Status and Coronary Artery Disease: A Cross Sectional Study

**DOI:** 10.5812/ircmj.13841

**Published:** 2014-03-05

**Authors:** Behrooz Ghanbari, Shiva Khaleghparast, Behshid Ghadrdoost, Hooman Bakhshandeh

**Affiliations:** 1 Mental Health Research Center, Tehran Psychiatric Institute, Iran University of Medical Sciences, Tehran, IR Iran; 2 Center for Nursing Care Research, Iran University of Medical Sciences, Tehran, IR Iran; 3 Shahid Rajaie Cardiovascular, Medical and Research Center, Iran University of Medical Sciences, Tehran, IR Iran

**Keywords:** Tea, Meat, Cardiovascular Diseases

## Abstract

**Background:**

Nutrition is among the most important factors influencing coronary artery disease.

**Objectives:**

Here we aimed to study the nutritional status of patients with and without coronary artery disease (CAD).

**Patients and Methods:**

We performed a cross-sectional study on 600 patients referred to a cardiology clinic with the signs of ACS. The patients were then classified in to two groups (CAD group and the normal group) based on angiographic findings. The amount of nutritional profile was questioned from all participants.

**Results:**

Men were more often diagnosed with CAD compared to women (198/362 vs. 102/238; P < 0.01). Patients with coronary artery disease were mostly older, smoker, coffee and black tea drinker had a higher BMI and more frequently diagnosed with hypertension, hyperlipidemia and diabetes. On the other hand, green tea consumption was seen more in women (92/238 vs. 115/362; P < 0.05) and those with regular physical activity (119/299 vs. 88/301; P < 0.01). Backward regression modeling was employed to study the predictors of CAD. Type of tea and meat remained as one the most important nutritional factors predicting CAD.

**Conclusions:**

White mean and type of tea were the most important predictors of CAD. Dietary prevention strategies from childhood could prevent early CAD.

## 1. Background

Nutritional status is the balance between the intake of nutrients by an organism and the expenditure of energy in the processes of growth, reproduction, and health maintenance. Because this process is highly complex and quite individualized, nutritional status assessment can be directed on a wide variety of nutrients from nutrient levels in the body, to the products of their metabolism, and the functional processes they regulate. Here are limited findings available on coronary artery disease (CAD) risk factors and nutritional pattern of CAD patients in Iran (1).

Recent studies have shown the role of calcium, magnesium, folate, and vitamins D and E in patients with coronary artery disease (2). Low levels of B12 contribute to the higher incidence of cerebrovascular disease and peripheral vascular disease, and low folate levels can lead to higher prevalence of hyperhomocysteinemia in coronary artery disease and cerebrovascular disease (3, 4) Administration of 1 g/d of omega-3 (EPA+DHA) in the form of fish oil can prevent sudden death in patients with acute coronary syndrome and can also help to reduce the number of hospital admission due to cardiovascular events in patients with chronic heart failures (5, 6). 

## 2. Objectives

The purpose of this study was to compare nutritional-related risk factors in patients with CAD that of matched controls in Iran.

## 3. Patients and Methods

We designed this cross-sectional study to evaluate the nutritional status in patients with coronary artery disease and matched controls. All the patients referred to the Rajaie Hospital for coronary angiography during March 2010 to December 2011. CAD was suspected according to the criteria of American College of Cardiology (7). The patients with CAD in angiography were considered as cases, and those without disease were considered as controls. Exclusion criteria were, history of angiography, revascularization therapy, cardiomyopathy, myocarditis, significant valvular disease, serum troponin I > 0.11, hemodynamic instability and congestive heart failure. Demographic and anthropometric data including age, gender, cigarette smoking, family history of coronary heart disease or diabetes, hypertension and hyperlipidemia were recorded. The blood pressure of the patients was measured two times after 5 minutes apart. The BMI (kg/m^2^) was calculated according to Quetelet formula. Participants, who consumed green tea during the past 12 months, were defined as “Green Tea user”. Also they were asked about the frequency, type of tea, age of initial tea consumption, amount of tea consumed each time, and duration of the consumption. “Physical Activity” were defined as aerobic physical activities more than 30 minute (walking, riding bicycle, running, and swimming) at least once a week. All patients were asked about their daily stress.

All the patients received Aspirin 325 mg and 300 mg of Clopidogrel. The patients then underwent coronary angiography. Indications for PCI, CABG, or medical therapy were made by the physician after reviewing the coronary angiography on the basis of clinical and para-clinical characteristics. All participants gave written informed consent before participation. The research was carried out according to the principles of the declaration of Helsinki; the local ethics review committee of Tehran University of Medical Science approved the study protocol.

### 3.1. Coronary Angiography

All patients underwent catheterization using standard Jenkins or Songs' techniques. Angiographic scoring was performed by two cardiologists. Coronary angiographies were interpreted visually and were analyzed in two orthogonal views. Stenosis > 50% in the main pericardial vessel was regarded as significant CAD (8). We used Braun Wald’s classification system to classify the patients according to their stenosis severity.

### 3.2. Statistical Analysis

The statistical package SPSS 17 for windows (Chicago, Illinois, USA), was used for analysis. Kolmogorov-Smirnov test was employed to test the normality of the variables in each group. Quantitative variables distributed normally are presented as mean ± standard deviation (SD). Qualitative variables are presented as number and percent. Chi square test was employed to measure the effect of green tea consumption on other variables. Kolmogorov Smirnoff test was employed to compare the amount of tea between patients with and without CAD. Backward logistic regression analysis was employed to study the important factors predicting CAD. The input variables were age, gender, BMI, educational level, physical activity, hypertension, hyperlipidemia, diabetes, smoking habits, coffee consumption, borage, herbal brew, type tea, diet, vegetable, dairy, fruit, nuts, red-meat, white-meat, egg, cereal, fat oil, olive oil, hydrogenated sunflower oil, corn, canola, others, mixed and stress. 

## 4. Results

Table 1 shows the primary characteristics of participants. Men were more often diagnosed with CAD compared to women (198:362 vs. 102:238; P < 0.01). There were significant difference between patients with and without CAD in respect to height, weight and BMI. Green tea was consumed more by women (92:238 vs. 115/362: P < 0.05) and more in those with regular physical activity (119:299 vs. 88:301; P < 0.01).

Then we compared the amount and type of nutrition in patients with and without CAD. Patients with Coronary artery disease were older, smoker, coffee and black tea drinker had a higher BMI and more frequently diagnosed with hypertension, hyperlipidemia and diabetes (Table 1). 

**Table 1.** Comparing the Primary Characteristics and Nutritional Status In Patients With and Without CAD ^a, b^

Variables	Patients without CAD (n = 152)	Patients with CAD (n = 251)	P Value
**Age **	47.9 ± 14.8	61.1 ± 11.6	< 0.01
**BMI**	26.9 ± 4.9	27.2 ± 4.2	< 0.001
**Education**			NS
Illiterate	65	168	
Diploma	37	61	
High school	50	22	
**CAD history in family**	47	100	< 0.01
**Physical activity**	96	109	< 0.01
**Hypertension**	37	121	< 0.001
**Hyperlipidemia**	38	123	< 0.001
**Diabetes**	23	74	< 0.001
**Smoking**	23	61	< 0.01
**Coffee**	53	54	< 0.001
**Type of tea**			< 0.001
green	16	10	
black	88	182	
mixed	48	59	
**Red meat**	137	228	NS
**White meat**	149	244	NS
**Stress**			NS
No	20	39	
Yes	54	91	
Occasionally	78	121	

^a^ Some of data are presented as mean ± SD

^b^ Abbreviations: BMI, body mass index; CAD, coronary artery disease

### 4.1. Predictors of CAD

Backward regression modeling was employed to study the predictors of CAD. After 21 steps of removing variables, type of tea and meat remained as one the most important nutritional factors predicting CAD (Table 2). 

**Table 2.** Predictors of Coronary Artery Disease in a Backward Regression Model

Variables	B	P Value	OR	Lower	Upper
**Gender, female**	-1.081	< 0.001	0.339	0.199	0.579
**Age, y**	0.075	< 0.001	1.078	1.055	1.101
**Educational level**					
Illiterate	0.986	0.009	2.680	1.283	5.601
Diploma		0.018			
High school	1.038	0.010	2.824	1.282	6.221
**Family History of CAD (Negative)**	-0.773	0.005	0.462	0.27	0.788
**Hypertension**	-0.57	0.044	0.566	0.324	0.987
**Diabetes**	-0.597	0.073	0.550	0.287	1.057
**Type of tea**		0.001			
Green tea	-1.41	0.010	0.244	0.083	0.715
Black tea	0.483	0.110	1.621	0.897	2.93
**White meat**	1.515	0.092	4.549	0.78	26.549

## 5. Discussion

Our findings from a population of patients with signs of acute coronary syndrome who were candidates of angiography showed that gender (Female) and negative family history of CAD are important factors in the prevention of CAD in these patients. On the other hand we did not found any significant difference between the studied groups in respect of dairy, vegetables, fruits, nuts, red and white meat, egg, cereal, fat oil, sunflower oil and canola. It could be questioned that while corn and olive oil were significantly different among the groups of patients with and without CAD, they did not had any value in the predication of CAD.

The findings of the current study confirmed the findings of the previous studies (9-11). In the later part of the 20th century, several studies had revealed that the consumption of olives in the Mediterranean diet is linked to a reduced incidence of CAD (12). The flavones classes were most strongly associated with lower risk of CVD (13). Vegetables have been shown to have important role in preventing CVD (14). Contrary to many studies have shown the importance of white meat and fish on CVD disease (15-17), in our study white meat was not significantly different among the groups of patients with and without CAD, which may be due to the difference in the study population.

Population-based studies have shown the beneficial effects of green tea on cardiovascular disorders (18), and reduced mortality rate of cardiovascular disorders in patients who consume green tea, but it does not have any effect on reducing the mortality rate of cancers (19). Studies have shown that the active components of green tea may result into oxidative challenge, and is against reactive oxygen species. They also reduce the LDL oxidizability. Besides they have lipid lowering effects. It is likely that green tea or its catechins lessen the absorption and tissue accumulation of other lipophilic organic compounds. It also prevents the metabolic syndrome. On the other hand, it has beneficial effects on vascular functionality (20). Consistently our study green tea provides a protective effect o against cardiovascular disorders. In many epidemiologic studies such as Amani's study, tea consumption has been a protective factor against CAD, but in this study black tea did not show such properties. This difference may be due to the difference in the study population and also other confounding factors. The principal limitation of the present study is its cross-sectional nature which precludes the determination of direct causality; however we took advantage of a relatively large sample size and close similarity between groups in most of the potentially confounding variables. In conclusion we show the beneficial effect of green tea consumption on preventing the CAD.
